# A Stress Surveillance System Based on Calcium and Nitric Oxide in Marine Diatoms

**DOI:** 10.1371/journal.pbio.0040060

**Published:** 2006-02-21

**Authors:** Assaf Vardi, Fabio Formiggini, Raffaella Casotti, Alessandra De Martino, François Ribalet, Antonio Miralto, Chris Bowler

**Affiliations:** **1** Laboratory of Diatom Signalling and Morphogenesis, Ecole Normale Supérieure, Paris, France; **2** Laboratory of Cell Signalling, Stazione Zoologica Anton Dohrn, Villa Comunale, Naples, Italy; **3** Section of Molecular Cytology & Centre for Advanced Microscopy, University of Amsterdam, Kruislaan, Amsterdam, Netherlands; **4** Laboratory of Ecophysiology, Stazione Zoologica Anton Dohrn, Villa Comunale, Naples, Italy; University of North CarolinaUnited States of America

## Abstract

Diatoms are an important group of eukaryotic phytoplankton, responsible for about 20% of global primary productivity. Study of the functional role of chemical signaling within phytoplankton assemblages is still in its infancy although recent reports in diatoms suggest the existence of chemical-based defense strategies. Here, we demonstrate how the accurate perception of diatom-derived reactive aldehydes can determine cell fate in diatoms. In particular, the aldehyde (2*E*,4*E/Z*)-decadienal (DD) can trigger intracellular calcium transients and the generation of nitric oxide (NO) by a calcium-dependent NO synthase-like activity, which results in cell death. However, pretreatment of cells with sublethal doses of aldehyde can induce resistance to subsequent lethal doses, which is reflected in an altered calcium signature and kinetics of NO production. We also present evidence for a DD–derived NO-based intercellular signaling system for the perception of stressed bystander cells. Based on these findings, we propose the existence of a sophisticated stress surveillance system in diatoms, which has important implications for understanding the cellular mechanisms responsible for acclimation versus death during phytoplankton bloom successions.

## Abstract 2

Aldehydes released by marine diatoms trigger intracellular calcium transients and nitric oxide generation, which results in cell death.

## Introduction

Diatoms are major components of phytoplankton blooms in aquatic ecosystems and are central in the biogeochemical cycling of important nutrients such as carbon, nitrogen, and silicon [1,2]. Unraveling the factors that regulate the fate of blooms is therefore of great importance. During a bloom succession, phytoplankton are thought to utilize chemical signals to enhance their defense capacities against grazers [3] and pathogens [4,5], and for outcompeting other phytoplankton for available resources [6,7]. The evolutionary and ecological success of diatoms in the contemporary oceans might suggest that they utilize sophisticated mechanisms to monitor and adapt appropriately to changing environmental conditions [8]. Indeed, previous reports have implicated the role of a chemical defense based on diatom-derived aldehyde products of fatty-acid oxidation [9,10], which impair the normal development of grazers such as copepods and other invertebrates [11,12]. Furthermore, it has now emerged that these same aldehydes are toxic to the diatoms themselves and can trigger a process bearing the hallmarks of programmed cell death [13]. We therefore explored the hypothesis that they may function as infochemicals in the marine environment, and so we investigated how diatoms perceive and respond to diatom-derived antiproliferative aldehydes such as (2*E*,4*E/Z*)-decadienal (DD). DD was chosen as a model aldehyde because its reactive properties are currently being tested on various animal, plant, and unicellular systems [14–16].

## Results/Discussion

**Figure 1:**
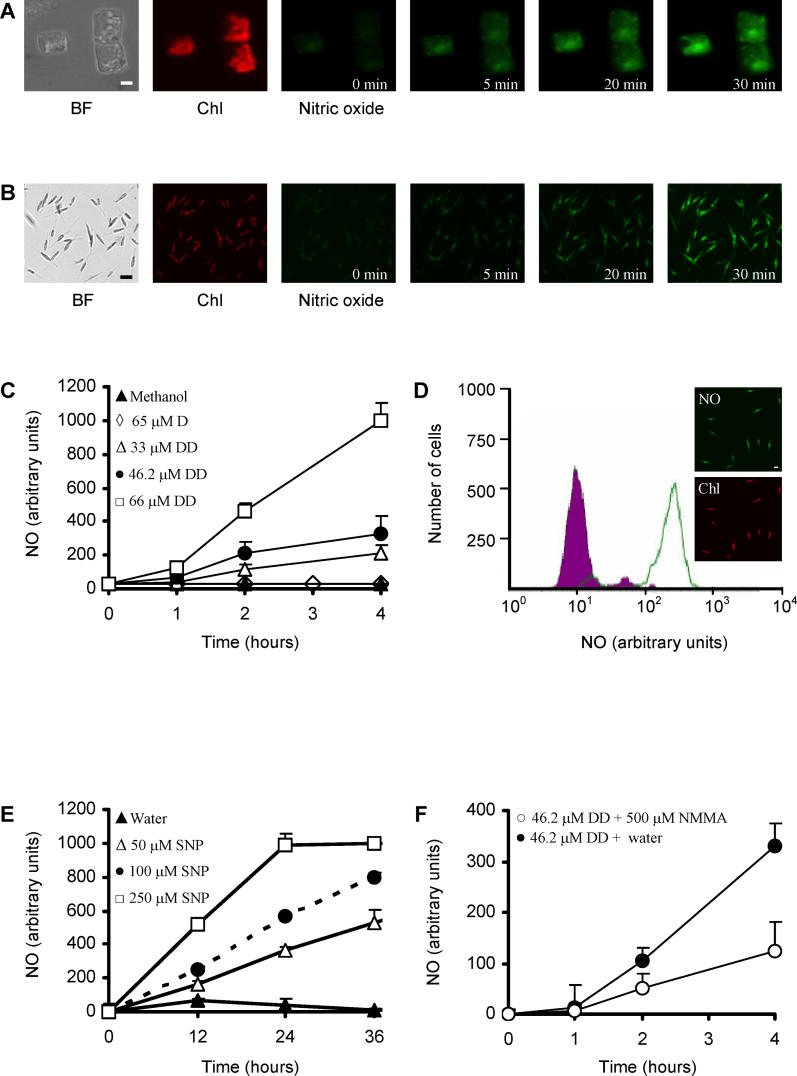
DD Induces NO Generation in Diatoms Micrographs depicting NO generation over time in response to DD (66 μM [10 μg/ml]) in T. weissflogii (A) and P. tricornutum (B). (C) Monitoring of NO production in P. tricornutum in response to a range of DD concentrations; (D) Cytogram showing NO generation 15 min after addition of DEANO (2 mM) to P. tricornutum cells (filled violet indicates the KOH control; open green indicates DEANO). Insets show epifluorescence micrographs of the DEANO-treated cells. (E and F) Relative accumulation of NO in P. tricornutum cells following treatment with SNP (E) or NMMA prior to exposure to DD (F). In all experiments, NO generation was assayed using the fluorescent probe DAF-FM. Data in (C), (E) and (F) are means plus standard deviation from four experiments. Representative data from at least four experiments are shown in (A), (B), and (D). Experiments shown in (C), (D), and (F) were performed by flow cytometry, and in (E) using a fluorescence microplate reader. BF, bright field; Chl, chlorophyll-derived red autofluorescence; D, (2*E*)-decenal. Scale bars represent 5 μm.

One of the early responses of plants and algae to pathogens and allelochemicals is thought to be the generation of reactive oxygen species (ROS) [7,17,18]. Our results indicated that DD did not stimulate detectable increases in general ROS production (assayed by dihydrorhodamine 123; data not shown), but rather induced the generation of nitric oxide (NO). NO exerts crucial physiological and developmental functions in both animals and plants, and is also involved in defense responses [19–21]. We monitored NO generation in two representative diatom species, *Thalassiosira weissflogii,* representing a cosmopolitan diatom genus, and *Phaeodactylum tricornutum,* which has become a central model for molecular and cellular studies of diatom biology [22,23]. Endogenous NO generation was measured by flow cytometry, fluorometry, and subcellular real-time imaging using the NO-sensitive dye 4-amino-5-methylamino-2′,7'-difluorofluorescein diacetate (DAF-FM) [24]. Microscopic analysis of T. weissflogii cells revealed that NO began to accumulate within 5 min after exposure to DD and increased significantly thereafter (Figure 1A). Furthermore, the DAF-FM fluorescence was localized close to the nucleus and was excluded from the plastid. A similar response was observed in P. tricornutum cells, in which the NO burst was also detected 5 min after exposure to DD (Figure 1B). In these short-term experiments, production of NO was proportional to DD concentration (between 33–66 μM [5–10 μg/ml]) with respect to the percentage of DAF-FM–positive cells, the extent of DAF-FM staining, and the lag time until significant numbers of cells emitted green fluorescence (Figure 1C). Treatments with methanol (1%), acetaldehyde (247 μM [10 μg/ml]), and other C10-unsaturated aldehydes such as (2*E*)–decenal (65 μM [10 μg/ml] failed to induce NO production (Figure 1C).

We used two NO donors, diethylamine nitric oxide (DEANO) and sodium nitroprusside (SNP), as positive controls to verify the reliability of DAF-FM as a probe for NO detection in P. tricornutum cells (Figure 1D and 1E). To further demonstrate DD-dependent NO production, we treated P. tricornutum cells with the NO synthase (NOS) antagonist *NG*-monomethyl-L-arginine (NMMA) prior to addition of DD (Figure 1F). This inhibitor reduced significantly the production of NO, implicating the possible involvement of NOS-like activities in NO generation (see below).

**Figure 2:**
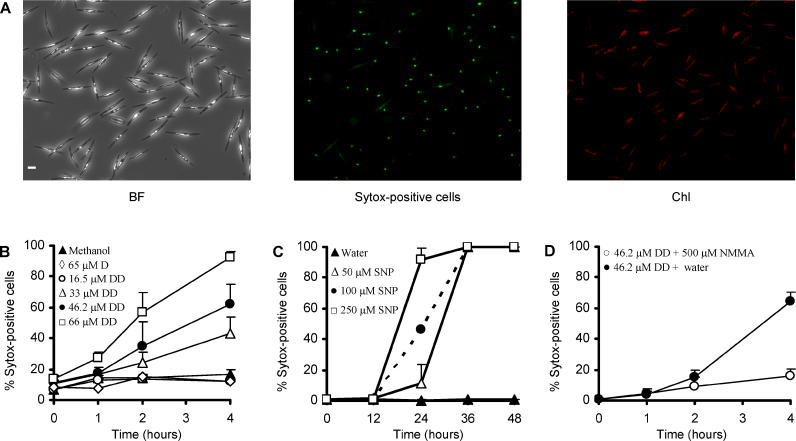
DD-Dependent NO Production Induces Cell Death (A) Micrographs of P. tricornutum cells treated with DD (66 μM [10 μg/ml]) for 4 h, which resulted in 90% cell death (assayed by Sytox Green fluorescence). Chlorophyll autofluorescence (shown in red) was significantly reduced in Sytox-positive cells, giving a further indication of cell death. (B–D) Quantification of cell death kinetics induced by DD or (*2E*)-decenal (B), SNP (C), and NMMA added prior to DD application (D). Data in (B–D) are means plus standard deviation from four experiments. Representative data from four experiments are shown in (A). Experiments shown in (B–D) were performed by flow cytometry. Abbreviations are as in Figure 1. Scale bar represents 5 μm.

A recent study in T. weissflogii showed that DD causes cell cycle arrest and induction of cell death, which was accompanied by morphological hallmarks of apoptosis [13]. Similarly, treatment of P. tricornutum cells with DD for 4 h led to cell death in more than 90% of the population, as evidenced by assaying plasma membrane integrity with the fluorescent dye Sytox Green, which is commonly used to detect dead cells [13,25] (Figure 2A). We further analyzed the kinetics of diatom cell death in response to a range of DD concentrations using flow cytometry (Figure 2B). DD was found to induce cell death in a dose- and time-dependent manner, and increased dramatically above a distinct threshold below which, although cell division was arrested, no cell death occurred. In these short-term experiments using cell densities of 2 × 10^5^ cells/ml, the threshold concentration of aldehyde required to induce cell death was around 19.8 μM (3 μg/ml). Treatments with methanol (1%), acetaldehyde (247 μM [10 μg/ml]) and (2*E*)-decenal (65 μM [10 μg/ml]) failed to induce significant cell death (Figure 2B).

To further examine the role of NO in determining diatom cell fate we examined cell death in response to an NO donor in the absence of DD, and treated cells with a NOS inhibitor prior to exposure to DD (Figure 2C and 2D). Treatment with the NO donor SNP led to an increase in the number of Sytox-positive cells, which coincided proportionally with NO accumulation (Figures 2C and 1E), in agreement with the threshold nature of the response to a range of DD concentrations (Figure 1C). Conversely, the NOS inhibitor NMMA could reduce DD-dependent cell death (Figure 2D). These data implicate the involvement of NO in the cell death cascade.

**Figure 3:**
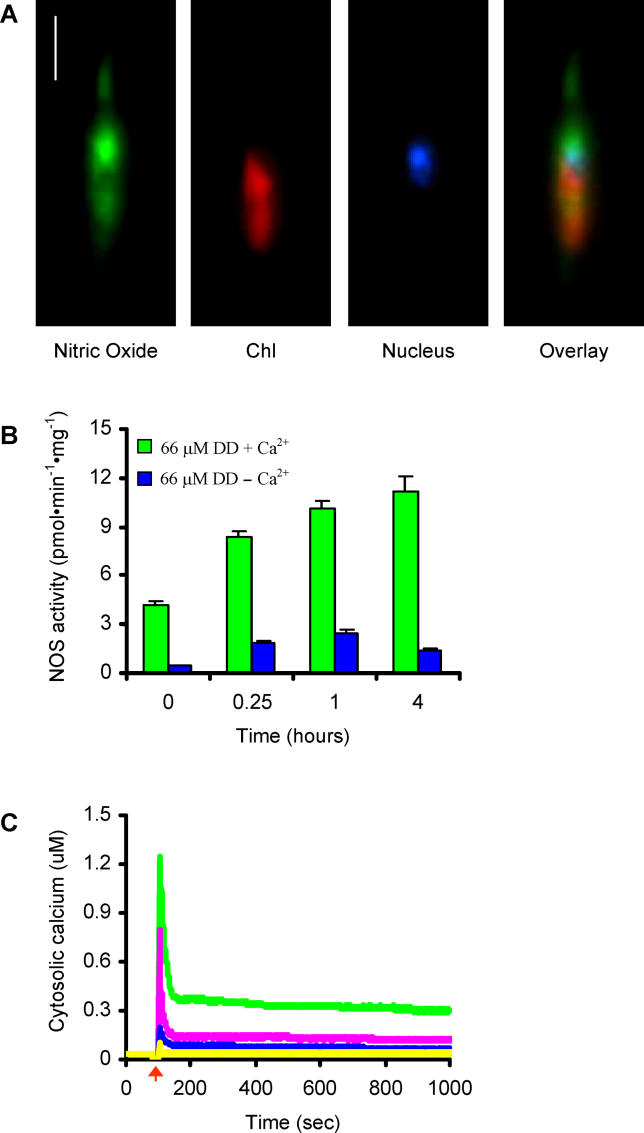
The Origin of NO in P. tricornutum and Its Interplay with Calcium (A) Intracellular localization of DAF-FM-derived fluorescence (green) compared with DAPI-staining (blue) and chlorophyll (Chl) autofluorescence (red) in P. tricornutum. (B) NOS enzymatic activity in cell-free extracts induced by DD (66 μM [10 μg/ml]), in the presence or absence of calcium. (C) Ca^2+^ transients in response to addition of 1, 3, and 5 μg/ml (6.6, 19.8, and 33 μM) DD, depicted in blue, pink, and green respectively and of 10 μg/ml (65 μM) (*2E*)-decenal (yellow) in transgenic P. tricornutum cells expressing the calcium-sensitive photoprotein Aequorin. Addition is indicated by arrow. Data in (B) are means plus standard deviation from four experiments. Representative data from at least four experiments are shown in (A) and (C). Scale bar represents 5 μm.

To investigate the intracellular origin of NO, we double stained P. tricornutum cells with DAF-FM and 4′,6-diamidino-2-phenylindole (DAPI) to label the nucleus (Figure 3A). Analysis of the images acquired by fluorescence microscopy showed that DAF-FM–derived fluorescence localized in neither the chloroplasts nor the nucleus, although it was closely associated with the latter. This could suggest that NO accumulates within a specific subcellular compartment, although one should caution that this observation could be a consequence of dye localization (DAF-FM fluorescence is nonetheless pH insensitive [24]). To further decipher the source of NO in diatoms, we assayed diatom extracts for NOS enzymatic activity using a conventional citrulline/arginine assay [26]. Basal NOS activity was 4 pmol • min^−1^ • mg^−1^ and increased significantly around 2.5-fold within the first 15 min after exposure to DD (Figure 3B). Analysis of the whole genome sequence of the diatom Thalassiosira pseudonana [27] (http://genome.jgi-psf.org/thaps1/thaps1.home.html, as well as the draft genome sequence of *P. tricornutum,* revealed several candidate genes with homology to genes encoding NO-generating enzymes from bacteria and plants [26,28–30], of which the diatom ortholog of the plant enzyme AtNOS1 (present in both diatom genomes) appeared to be the most likely candidate, based on overall similarity (data not shown). Indeed, diatom extracts exhibited NOS activity that was similar to the plant NOS enzyme [31] in that activity was strongly calcium dependent (Figure 3B).

Calcium is known to be an important second messenger for a wide variety of environmental stimuli in both plant and animal cells [32,33]. Previous studies have revealed that P. tricornutum displays sophisticated sensing systems for perceiving abiotic environmental signals that involve calcium-dependent signal transduction mechanisms [34]. We used transgenic P. tricornutum cells expressing the calcium-sensitive photoprotein Aequorin to detect transient changes in cytosolic calcium in response to reactive aldehydes. Application of DD stimulated a dramatic increase in intracellular calcium that persisted for several minutes before returning to basal levels, whereas its monounsaturated form, (2*E*)-decenal, or methanol, its solvent, did not provoke any substantial response (Figure 3C). As seen both for DD-dependent NO production and cell death, DD triggered Ca^2+^ release with maximal amplitude proportional to the applied dose (Figure 3C). In an attempt to identify the source of the cytosolic calcium increase, we exposed P. tricornutum cells to the impermeant form of BAPTA (1,2-Bis(2-aminophenoxy)ethane-N,N,N′,N′-tetraacetic acid, tetrapotassium salt), a known highly selective calcium chelating reagent, prior to addition of DD. This chelator had no effect on the DD-dependent calcium transient, but suppressed the cellular response to hypo-osmotic shock (Figure S1). These data suggest that internal calcium stores are responsible for the Ca2+ release in response to DD, contrasting with the external origin of cytosolic calcium induced in response to the abiotic stress.

To our knowledge this is the first time that NO has been detected in marine phytoplankton, although it has been detected in sea water and was suggested to originate from abiotic nitrite photolysis and from bacterial denitrification/nitrification cycles [35,36]. Neither short- nor long-term exposures to NO donors (DEANO and SNP) led to any detectable increases in cytosolic Ca^2+^ (data not shown), suggesting that NO acts downstream of Ca^2+^ in the signaling cascade, in agreement with the earlier response of calcium compared with NO following addition of DD, and the Ca^2+^ dependency of NOS activity (Figures 1B, 3B, and 3C). Furthermore, several control compounds (see above) failed to induce either calcium transients or NO production, and did not induce cell death. Conversely, other pharmacological agents that amplified the calcium response (e.g., nifedipine) also amplified changes in NO and increased cell death (data not shown), implying a causal link between calcium and NO in the induction of cell death. Our results therefore suggest a signaling pathway in which DD-induced cell death in diatoms is preceded by accurate perception of the aldehyde, followed by changes in intracellular calcium that may activate a plant-type NOS to subsequently generate NO.

**Figure 4:**
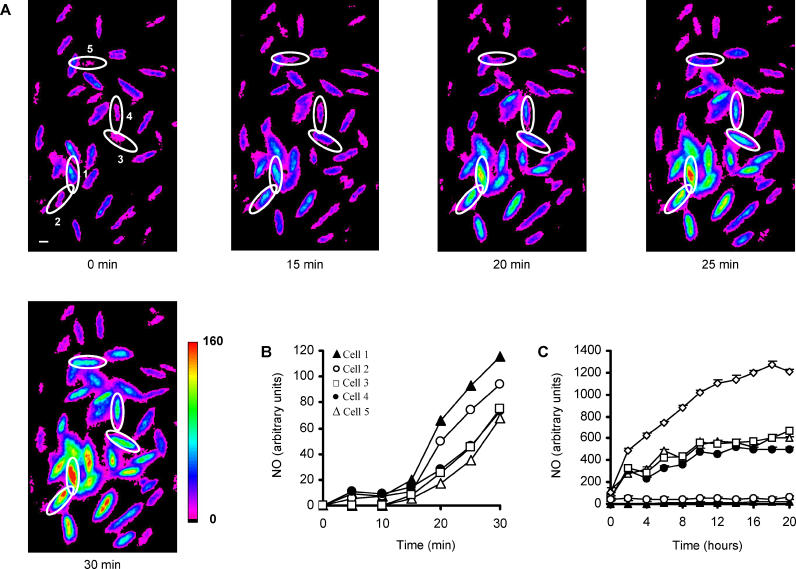
Intercellular Transmission of a DD-Derived Signal that Induces NO in Neighboring Cells (A and B) In vivo imaging of DD-induced NO burst in P. tricornutum cells. (A) Time course of NO production in single cells based on corresponding real-time movie (see Video S1). (B) Relative accumulation of NO in selected cells of the micrographs shown in (A). (C) NO accumulation in an untreated population of DAF-FM-loaded cells that were inoculated with a non DAF-FM–loaded population that had been exposed to DD at different concentrations (open circle, methanol solvent; open square, 660 nM; filled circle, 3.3 μM; open triangle 6.6 μM, and open diamond, 13.2 μM) for 24 h prior to the mixing. Incubation of fresh medium with 13.2 μM DD for 24 h (filled triangle) prior to addition to DAF-FM-loaded cells did not provoke any detectable increase in NO. Data in (C) are means plus standard deviation from four experiments.

Real-time imaging of NO generation in P. tricornutum cells treated with DD revealed that after 30 min, intracellular levels of NO were at least 10-fold higher in reacting cells with respect to basal levels (Figure 4A). Furthermore, some of the cells displayed higher sensitivity and responded to DD earlier than in adjacent cells (see Video S1). Neighboring cells in the proximity of these early-responding cells exhibited significantly delayed responses (Figure 4B), suggesting the generation of a diffusible NO-inducing signal from reacting cells. These observations suggested a DD-derived intercellular communication system that could propagate within the diatom population.

In order to examine this intercellular signaling phenomenon further, we designed an experiment in which cells were exposed to a range of DD concentrations (660 nM–13.2 μM [0.1–2.0 μg/ml]) for 24 h (population A) and then were mixed with DAF-FM–preloaded cells that were not exposed directly to DD (population B). Monitoring DAF-FM fluorescence in the untreated population B revealed that NO accumulated over the next 24 h (Figure 4C). Interestingly, the response correlated with the level of DD pretreatment of population A and was already detectable in response to cells treated with very low sublethal concentrations (e.g., 660 nM [0.1 μg/ml]). Furthermore, the pattern of NO production resembled a threshold response: Population A cells treated with DD concentrations between 660 nM and 6.6 μM (0.1–1.0 μg/ml) provoked equivalent NO production profiles in population B cells, whereas treatment with 13.2 μM (2.0 μg/ml) DD generated much higher changes in NO (Figure 4C). Such responses are in agreement with the EC50 value for P. tricornutum cell growth of 7.06 μM ± 2.44 μM (1.07 ± 0.37 μg/ml) calculated by Probit analysis for initial cell densities of 10^5^ cells/ml after exposure to DD for 24 h (data not shown). These data suggested that diatom cells could detect the level of stressed cells within the population by sensing a DD-derived diffusible signal (or signals) transmitted by wounded cells to neighboring healthy cells. To exclude the possibility that the observed response was simply due to the residual presence of DD in the medium, fresh cell free medium was incubated with the same DD concentrations for 24 h prior to addition to population B cells. Such treatments failed to induce any detectable increase in NO (Figure 4C), confirming that the aldehyde was likely to be degraded over the 24-h period [37].

A recent report [11] has demonstrated arrested larval development in copepods fed on dinoflagellates that were treated with DD concentrations in the range of 9.9 μM (1.5 μg/ml). In these experiments it was proposed that the dinoflagellates acted as DD carriers by absorption of the molecule to the cell surface. However, the amount of DD absorbed by the dinoflagellate carrier cells ranged from only 0.4 fg cell^−1^ to 36 fg cell^−1^, much lower than the total aldehyde concentration initially inoculated in the culture medium. Considering this finding and considering that even lower concentrations of DD (660 nM [0.1 μg/ml]) led to responses in our untreated population B cells, we propose that unsaturated aldehydes such as DD could play an important role in diatom assemblages during blooms, in which local densities can reach as high as 5 × 10^5^ cells/ml [38]. Benthic diatoms living in dense microbial mats and biofilms are also known to produce fatty acid derivatives [39]. Such microenvironments, consisting of high cell densities within polysaccharide matrices, are likely to further facilitate the potential for cell–cell communication and to further enhance defenses against grazers [40].

**Figure 5:**
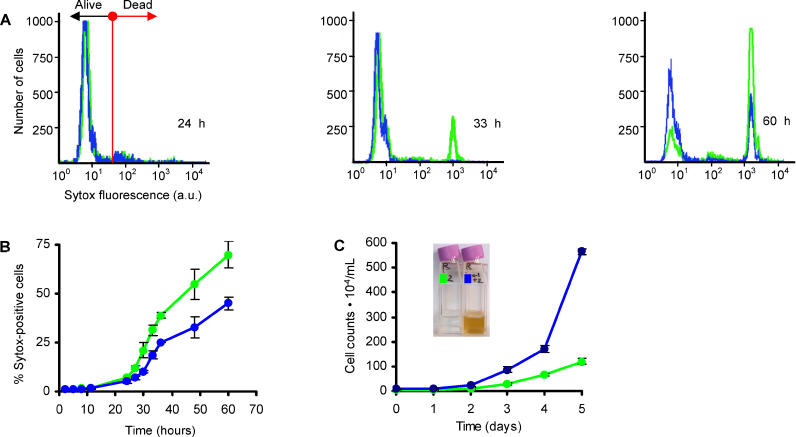
Sublethal DD Concentrations Can Induce Resistance to Lethal Doses in P. tricornutum Cells were pretreated with 0.1 μg/ml (660 nM) DD for 2 h prior to subsequent addition of 2 μg/ml (13.2 μM) DD (blue) and compared to a single dose treatment of 2 μg/ml (13.2 μM) DD (green). (A and B) Cell death was assayed by flow cytometry both qualitatively in cytograms (A) and quantitatively (B) using Sytox Green at the indicated time points. (C) Cell growth curves following resuspension of pretreated and non-pretreated cells in DD-free fresh medium 60 h after the 2 μg/ml (13.2 μM) DD treatment. The time scale indicates the days following resuspension. Inset shows photograph of the two cultures taken 2 wk after resuspension starting from an initial inoculum of 5 × 10^4^ cells/ml. Representative data from at least five experiments are shown in (A–C). a.u., arbitrary units.

Based on our observations presented in Figure 4, we further explored the signaling role of unsaturated aldehydes in diatom populations. We aimed to mimic conditions in which diatoms could be exposed to different doses of DD on successive occasions, as may be the case in natural environments. We performed long-term experiments in which cells were exposed to sublethal doses of DD (660 nM [0.1 μg/ml]) for 2 h prior to subsequent addition of a higher dose (13.2 μM [2 μg/ml]), and compared the responses to a single dose only. The two populations displayed remarkable differences. Substantial cell death was already visible after 33 h in the non-pretreated culture, whereas cell death in the pretreated culture was significantly delayed (Figure 5A). After 60 hr, cell death in the preconditioned population was around 40%, whereas more than 70% of cells in the non-preconditioned population were positively stained with the cell death indicator Sytox Green (Figure 5B). Furthermore, resuspension of the same cultures in DD-free fresh medium revealed a notable difference in growth rates, with a 6-fold increase in cell density in the acclimated population compared with cultures from non-acclimated cells after 5 d (Figure 5C). Interestingly the pretreated culture could fully recover, whereas the non-acclimated culture failed to be viable and ultimately collapsed (Figure 5C, inset). These data demonstrate the potential of DD as an infochemical for regulating cell fate in diatom populations at doses one order of magnitude lower than used in previous reports [10,11,13]. Specifically, it appeared that pretreatment with sublethal doses of DD could stimulate resistance to normally lethal concentrations.

**Figure 6:**
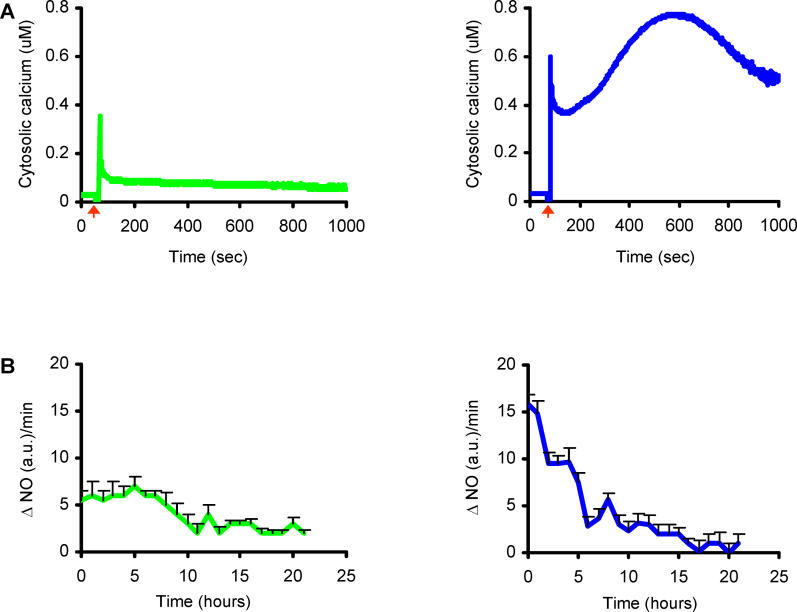
The Role of NO and Calcium in Acclimation to Sublethal Doses of DD (A) Cytosolic calcium transients in transgenic P. tricornutum cells expressing Aequorin. Left panel shows calcium response in non-pretreated cells (green) following addition of 13.2 μM (2 μg/ml) DD (indicated by arrow), whereas right panel shows the calcium signature of cells that had been pretreated with 660 nM (0.1 μg/ml) DD for 2 h prior to addition of 13.2 μM (2 μg/ml) DD (blue). (B) Relative rate of NO production in pretreated (blue) and non-pretreated (green) cells. Experimental conditions as in (A). Representative data from five experiments is shown in (A). Data in (B) are means plus standard deviation from five experiments. a.u., arbitrary units.

To examine the molecular mechanism responsible for these contrasting responses (induced resistance versus death), we examined the role of calcium and nitric oxide. Preconditioned P. tricornutum cells treated with 660 nM (0.1 μg/ml) DD for 2 h, which did not provoke any changes in intracellular calcium (data not shown), were dramatically sensitized to a successive administration of 13.2 μM (2.0 μg/ml) DD (Figure 6A). The initial peak in cytosolic calcium was increased almost 2-fold compared with cells that were not preconditioned, and a second more-sustained peak was apparent that was not seen in non-pretreated cells. Interestingly, this modulation in the calcium signature correlated with a 3-fold increased rate of NO production in the acclimated population during the first 2 h after exposure to the second treatment (Figure 6B). Only after 5 h did the rate of production return to steady-state levels that were similar in the two populations. Such a pronounced increase in the preconditioned population could be due either to a higher production rate of NO per cell or to a higher number of reacting cells, as also observed in the real-time studies of NO propagation within a DD-treated population (see Video S1). In either scenario this result implies that NO is associated not only with cell death but also with induced resistance. The results in Figure 4C confirm this, in that NO production increases both in response to lethal and sublethal DD concentrations. However, in both this and other experiments (see Figures 1–4) we observed a clear threshold response, suggesting that sublethal doses of DD may trigger signaling phenomena that lead to induced resistance, whereas higher doses induce cell death. In terms of calcium, the different signatures of cell populations destined to die and those with induced resistance may suggest that the first acute response may trigger active cell death, whereas the second sustained response may override this and induce resistance responses. The mechanisms whereby the same molecule can mediate such contrasting responses must be the subject of future study. Interestingly, a similar phenomenon has been reported for NO in plants [41].

In these studies we used the best-characterized unsaturated aldehyde, DD, to study effects on diatom cell-fate regulation, and responses to very low concentrations could be observed. However, previous reports demonstrate that a variety of unsaturated aldehydes are produced by different diatom species, and variability can also be found among different strains of the same species [10,42,43]. Hence, local concentrations of reactive aldehydes may be considerable and they may display synergistic effects not yet tested. Furthermore, analysis of the amount of aldehydes produced by different diatom species has revealed a high variability ranging between 30 and 869 fg per cell [11,44], and it appears that aldehyde production is continuous once cell membrane integrity is disrupted [9]. A variety of factors may therefore contribute to raise actual concentrations further, and so it is reasonable to believe that the observations reported here may have ecological relevance. Recent attempts to develop a sensitive method for the detection and quantification of diatom-derived aldehydes in cultures and in natural populations will clearly help determine the ecological role of these molecules in aquatic habitats [44].

In conclusion, our results demonstrate that diatom cells can sense local DD concentrations and integrate this information in a temporal context. Because aldehydes such as DD are released by wounded diatom cells [9], we propose that they are used as infochemicals to provide a surveillance system to evaluate stress during bloom conditions. Indeed, in chrysophytes, aldehyde concentrations also increase following exposure to abiotic stress (e.g., light and nutrients [45]). Furthermore, perception of some aldehydes could be used for the detection of other phytoplankton competitors, analogous to the cross talk (allelopathy) observed between a bloom-forming dinoflagellate and toxic *Microcystis* sp. in Lake Kinneret [7]. Perception of sublethal levels of aldehydes by cells in the locality of bystander damaged cells could sensitize calcium- and NO-based signaling systems to induce resistance to successive aldehyde exposure, providing an early-warning protective mechanism, as clearly observed in Figure 5. In a somewhat analogous fashion, plants use volatile organic compounds (VOC) for chemical communication to provide immunity during plant–plant–herbivore interactions [46].

When stress conditions aggravate during a bloom, and cell lysis rates increase, aldehyde concentrations could exceed a certain threshold, and may function as a diffusible bloom-termination signal that triggers population-level cell death [25,47]. It is now established that coordination of stress responses, cell survival, and death can also operate in unicellular organisms and can orchestrate multicellular-like behavior [48,49]. Based on our observations, we therefore propose that differential production and sensitivity to reactive aldehydes by diatoms may determine the fitness and succession of phytoplankton communities in the marine environment through mechanisms regulated by intracellular calcium and NO signals. Such a hypothesis is further supported by observations that different species and even different strains of the same species display qualitative and quantitative differences in aldehyde production [10,42,43].

A recent field study has evaluated intraspecies genetic variability in populations of the harmful diatom *Pseudo-nitzschia delicatissima* and indicated a high genetic variability in pre-bloom conditions whereas only one major clade dominated during the peak of the bloom [50]. Future studies should therefore determine the function of infochemicals such as unsaturated aldehydes in mediating selection in intra- and interspecies interactions during bloom succession.

## Materials and Methods

### Diatom growth conditions

Phaeodactylum tricornutum Bohlin strain CCMP 632 and Thalassiosira weissflogii clone CCMP 1336 were obtained from the Provasoli-Guillard National Center for Culture of Marine Phytoplankton (West Boothbay Harbor, Maine, United States). Transgenic lines of P. tricornutum expressing the *Aequorin* gene were obtained as previously described [34] and were grown axenically in artificial sea water (ASW) at 20 °C in a 12-h photoperiod (100 μmol • m^−2^ • s^−1^). T. weissflogii cells were grown axenically in filtered sea water (SW) enriched with nutrients as in f/2 medium. Exponentially growing cultures at cell densities from 1 × 10^5^ to 5 × 10^5^ cells/ml were used for all experiments.

### Chemicals

Dihydrorhodamine 123 (5 mg/ml stock in ethanol), coelenterazine (1 mM stock in methanol), DEANO (10 mM stock in KOH [pH 12]), DAF-FM (5 mM stock in DMSO), SNAP (*S*-nitroso-*N*-acetylpenicillamine; 100 mM stock in DMSO), NMMA (100 mM stock in water), DAPI (4′,6-diamidino-2-phenylindole [5 mg/ml stock in water]), impermeant BAPTA ((1,2-Bis(2-aminophenoxy)ethane-N,N,N′,N′-tetraacetic acid, tetrapotassium salt; 1M stock in MOPS [pH 7.2]), and Sytox Green nucleic acid stain (5 mM stock in DMSO) were purchased from Molecular Probes-Invitrogen (http://probes.invitrogen.com). SNP (sodium nitroprusside; 100 mM fresh stock in water), acetaldehyde, (2*E*)-decenal, and DD were obtained from Sigma-Aldrich (http://www.sigma-aldrich.com). DD from Sigma-Aldrich was used for all experiments except for Aequorin assays, in which we used a purified preparation of diatom-derived DD, kindly provided by Dr. Georg Pohnert (MPI, Jena, Germany). DD was dissolved in methanol, and concentrations were determined by measuring absorption at the lambda max for DD of 274 nm, using a Hewlett-Packard 8453 spectrophotometer (Hewlett-Packard Company, Palo Alto, California, United States).

### Fluorescence detection

Fluorescence microscopy was performed using the following filters from Omega Optical (http://www.omegafilters.com: XF104–2 (for DAF-FM and Sytox Green detection), XF39 (for chlorophyll detection), and XF06 (for DAPI detection). Image acquisition was performed using a Hamamatsu ORCA-100 CCD camera (Hamamatsu Photonics, Hamamatsu City, Japan). For the video (see Video S1), DAF-FM-loaded cells were embedded in 0.5% low melting point agarose (Bio-Rad, Hercules, California, United States) in ASW. Image acquisition in the microscope was begun 5 min after addition of DD (10 μg/ml), using a 20× objective and an intensified CCD camera, I-PentaMAX Gen III ICCD:HB, from Princeton Instruments (Roper Scientific, Tucson, Arizona, United States).

For NO measurements, *P. tricornutum* or T. weissflogii cells were incubated in the dark with 10 μM DAF-FM for 60 min followed by two washing steps (incubation for 30 min after the first wash to allow de-esterification). Efficiency of loading was tested by examining DAF-FM–dependent fluorescence in the microscope following addition of the NO donor SNAP (0.5 mM). To quantify NO accumulation, DAF-FM fluorescence was measured either with a Bio-Tek FL600 Fluorescence Microplate Reader using a GFP filter set (excitation 485/30, emission 530/30), or using a FACScalibur Becton-Dickinson flow cytometer (Becton-Dickinson, Palo Alto, California, United States) equipped with a 488-nm laser as excitation source. A 530/30BP emission filter was used for detection of DAF-FM–derived fluorescence. Cell death was assayed using Sytox Green [13,25], and fluorescence was monitored both microscopically and using flow cytometry or a fluorescence microplate reader, as for NO.

### Determination of NOS activity

DD-treated cells were harvested and sonicated with lysis buffer (10 mM Tris-HCl [pH 7.5], 0.5 mM EDTA, 50 mM NaCl, 1 mM DTT, 0.1% Triton X-100, and protein inhibitor cocktail). The protein solution was then used to measure NOS activity with a NOS assay kit from Cayman Chemicals (http://www.caymanchem.com) as described in Guo et al. [26]. In order to determine whether the reaction was calcium dependent, 5 mM EDTA was added prior to the assay. Protein quantification was determined using the Bio-Rad Lowry kit.

## Supporting Information

Figure S1 DD Triggers Calcium Release from Internal Stores P. tricornutum cells were treated with impermeant BAPTA (50 mM) prior to exposure to either DD (A) or a hypo-osmotic shock (25% ASW) (B). Traces from BAPTA-treated cells are in blue, traces from untreated cells are in green.
(46 KB PDF).

Video S1 In Vivo Imaging of DD-Induced NO Burst in P. tricornutum Cells Real-time movie of DAF-FM–loaded *P. tricornutum* cells treated with 66 μM (10 μg/ml) DD and imaged for 35 min for NO detection. Frame interval: 10 s. Movie time: approximately 35 min (QuickTime).
(3.2 MB MOV).

## Abbreviations

DAF-FM: 4-amino-5-methylamino-2′,7'-difluorofluorescein diacetate
DAPI: 4′,6-diamidino-2-phenylindole
DD: (2*E*,4*E*/*Z*)-decadienal
DEANO: diethylamine nitric oxide
NMMA: *N*G-monomethyl-L-arginine
NO: nitric oxide
NOS: nitric oxide synthase
ROS: reactive oxygen species
