# Supplementary material for: A Stress Surveillance System Based on Calcium and Nitric Oxide in Marine Diatoms
Source: PLoS Biol. 2006 Feb 21;4(3):e60. doi: 10.1371/journal.pbio.0040060 (PMC1370914; doi:10.1371/journal.pbio.0040060)
Supplement: Supplementary file 1 [file pbio.0040060.sg001.pdf]

Fig. S1

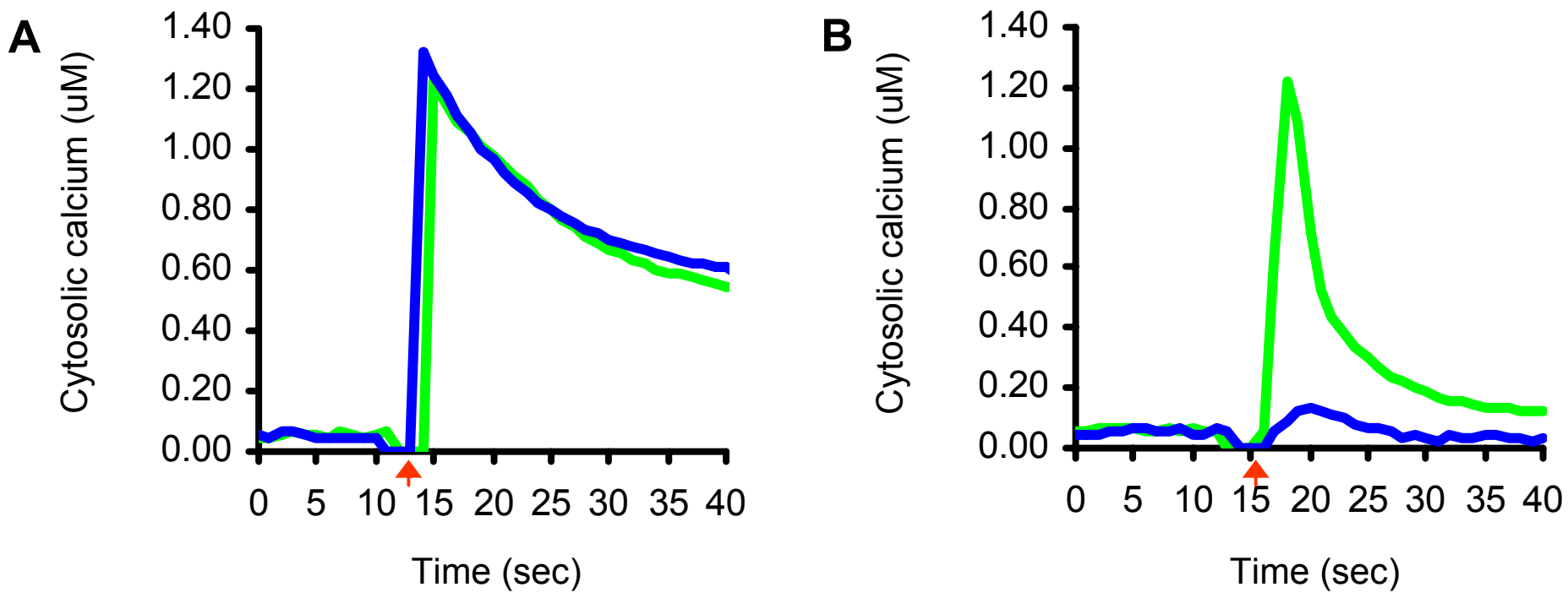
A
B
Cytosolic calcium (uM)
1.40
1.20
1.00
0.80
0.60
0.40
0.20
0.00
0 5 10 15 20 25 30 35 40
Time (sec)
